# Flower-like CoFe-LDH Activated Peroxymonosulfate for Tetracycline Degradation: Efficiency and Mechanism

**DOI:** 10.3390/toxics14050389

**Published:** 2026-04-30

**Authors:** Yiting Luo, Yihui Zhou, Tao Xu, Rongkui Su, Xiancheng Ma, Wende Yan

**Affiliations:** 1 Hunan First Normal University, Changsha 410114, China; 2 College of Life and Environmental Science, Central South University of Forestry and Technology, Changsha 410004, China; 3 School of Vehicle Application, Hunan Automotive Engineering Vocational University, Zhuzhou 412001, China

**Keywords:** tetracycline, CoFe-LDH, peroxymonosulfate, advanced oxidation

## Abstract

The overuse of antibiotics has led to their widespread environmental residues, posing a significant threat to the ecological environment. In this study, a flower-like spherical CoFe-layered double hydroxide (CoFe-LDH) catalyst was prepared using a hydrothermal method. The degradation performance of the CoFe-LDH/peroxymonosulfate (PMS) system was systematically investigated using tetracycline (TC) as a model pollutant. The CoFe-LDH exhibited a three-dimensional nanoflower-like spherical structure formed by interlaced nanosheets, featuring smooth surfaces and well-defined edges. This hierarchical porous structure facilitates the exposure of active sites. The CoFe-LDH/PMS system demonstrated remarkable degradation efficiency, achieving over 90.17% TC removal within 10 min. As the dosage of CoFe-LDH and PMS increases, the degradation rate of TC improves significantly, but the marginal improvement effect decreases. TC degradation efficiency increased with pH up to an optimum at pH 5.0, beyond which it declined. The anions—Cl^−^, $NO_{3}^{-}$, and $SO_{4}^{2-}$—all exhibited inhibitory effects on TC degradation; the TC removal rates decreased to 77.88%, 80.58%, and 82.78%, respectively. The removal experiments of different organic pollutants, such as oxytetracycline (88.91%), methylene blue (98.36%), and ciprofloxacin (84.52%), as well as actual water experiments, such as lake water (92.48%) and tap water (80.86%), have demonstrated the good universality of the CoFe-LDH/PMS system. Radical quenching experiments confirmed that ^•^OH and $SO_{4}^{•-}$ were the dominant reactive species.

## 1. Introduction

The widespread use of antibiotics in healthcare, agriculture, and animal husbandry has been effective in controlling diseases [1,2]. However, the ecological risks arising from their irrational use also pose a global public health challenge [3,4]. The misuse and overuse of antibiotics can lead to a marked increase in drug residues in environmental matrices, resulting in intergenerational cumulative toxic effects on ecosystems and human health [5,6,7]. As one of the most widely used classes of antimicrobial agents in clinical practice, tetracycline antibiotics (including tetracycline, oxytetracycline, chlortetracycline, and doxycycline, among others) persist in the environment [8,9,10]. Their prolonged residual presence not only significantly alters the diversity and community structure of microorganisms at the soil-water interface but also enters human exposure pathways through environmental transport [10,11,12]. Therefore, there is an urgent need to explore efficient, green technologies for controlling tetracycline antibiotic pollution.

The progressive development of free radical-driven oxidation technologies has provided a core driving force for the efficient treatment of emerging pollutants in aquatic environments [13,14]. Kalidhasan et al. reported the synthesis, characterization, and application of supramolecular polymer–montmorillonite clay–copper oxide (MPC) as an efficient catalyst for the degradation of rhodamine B. The results showed that MPC can catalyze H_2_O_2_ to generate hydroxyl radicals (^•^OH) active species for the rapid removal of rhodamine B [15]. Compared to conventional advanced oxidation processes (AOPs) based on ^•^OH, the new generation of persulfate-based AOPs (PS-AOPs) exhibits superior adaptability for environmental applications [16,17,18]. Their key advantage lies in the synergistic evolution of multiple reactive species, which effectively overcomes dependence on reaction pH and enables deep degradation of organic pollutants by virtue of a significantly extended oxidant half-life [19,20,21]. Currently, persulfate activation strategies have expanded from homogeneous systems (e.g., light, heat, transition metal ions) to heterogeneous catalytic systems [22,23,24,25]. The latter have become a research frontier due to their advantages in catalyst recyclability, high stability, and low risk of secondary pollution [26,27,28]. However, the performance enhancement of heterogeneous catalytic systems ultimately depends on the intrinsic activity and stability of the catalyst [29,30].

Layered double hydroxides (LDHs) are nanomaterials with highly tunable chemical compositions and layered structures [31,32,33]. They consist of mixed-valence metal hydroxides, interlayer anions, and water molecules arranged in stacked layers [34,35,36]. The types and molar ratios of the metal elements, the species and amount of interlayer anions, and the stacking mode collectively determine the primary properties of LDHs [37,38,39]. Owing to their flexible composition, high stability, anion exchange capacity, large surface area, memory effect, and environmentally friendly characteristics, LDHs can serve as high-performance adsorbents or catalysts [40,41,42]. For instance, Zhang et al. synthesized a calcined CuBi_2_O_4_/ZnAlBi-LDHs composite using an ultrasound-assisted calcination method, which exhibited excellent adsorption and photocatalytic performance for the simultaneous removal of Cr(VI) and TC, achieving removal efficiencies exceeding 95% [43]. Nevertheless, the role of LDHs in catalyzing persulfate systems warrants further investigation.

The unique layered structure of cobalt iron layered double metal hydroxide (CoFe-LDH) can endow the material with a large specific surface area and abundant active sites. The layered defect structure can also promote the transport of proton acceptors (such as OH^−^), which is beneficial for catalytic reactions [44]. In this study, a flower-like spherical cobalt-iron layered double hydroxide (CoFe-LDH) was prepared via a hydrothermal method. The morphological features, chemical composition, and structural characteristics of the as-prepared CoFe-LDH were systematically characterized. An advanced oxidation process based on persulfate activation was subsequently established using CoFe-LDH as the catalyst. The kinetic behavior, performance, and underlying mechanisms of tetracycline (TC) degradation in the CoFe-LDH/persulfate system were comprehensively investigated.

## 2. Materials and Methods

### 2.1. Reagents and Instruments

Cobalt(II) nitrate hexahydrate (Co(NO_3_)_2_·6H_2_O, 99%), iron(III) nitrate nonahydrate (Fe(NO_3_)_3_·9H_2_O, analytical grade), urea (CH_4_N_2_O, analytical grade), ammonium fluoride (NH_4_F, analytical grade), and potassium peroxymonosulfate (HKSO_6_, analytical grade) were purchased from Shanghai Macklin Biochemical Co., Ltd. (Shanghai, China). Methanol (MeOH, analytical grade), tert-butanol (C_4_H_10_O, analytical grade), hydrochloric acid (HCl, analytical grade), sodium hydroxide (NaOH, analytical grade), potassium chloride (KCl, analytical grade), potassium nitrate (KNO_3_, analytical grade), and potassium sulfate (K_2_SO_4_, analytical grade) were obtained from Sinopharm Chemical Reagent Co., Ltd., Shanghai, China. Tetracycline hydrochloride (C_22_H_24_N_2_O_8_, ≥88.5%) was purchased from Solarbio Science and Technology Co., Ltd. (Beijing, China).

A digital magnetic stirrer (ZGCJ-3A, Shanghai Zigui Instrument Co., Ltd., Shanghai, China), an ultrapure water system (UPT-11-40, Chengdu UP Instrument Co., Ltd., Chengdu, China), a vacuum drying oven (DZ-2BCIV, Tianjin Taisite Instrument Co., Ltd., Tianjin, China), a benchtop high-speed centrifuge (TG16-WS, Hunan Xiangyi Centrifuge Instrument Co., Ltd., Changsha, China), an electronic balance (DHG-9023A, Shanghai Precision Laboratory Equipment Co., Ltd., Shanghai, China), a digital pH meter (PHS-3E, Shanghai Yidian Scientific Instrument Co., Ltd., Shanghai, China), a Fourier transform infrared (FTIR) spectrometer (NICOLET iS20, Thermo Scientific, Waltham, MA, USA), an X-ray diffractometer (MiniFlex600, Rigaku, Tokyo, Japan), a scanning electron microscope (JSM-7610FPlus, Jeol, Tokyo, Japan), a UV–Vis spectrophotometer (UV-2600, Shimadzu, Tokyo, Japan), and a micropipette (100–1000 μL, Thermo Scientific, Waltham, MA, USA) were used in this study.

### 2.2. Material Preparation and Characterization

Cobalt(II) nitrate hexahydrate (3.2 mmol) and iron(III) nitrate nonahydrate (0.8 mmol) were added to 30 mL of ultrapure water and stirred for 30 min to form Solution A. Meanwhile, urea (10 mmol) and ammonium fluoride (10 mmol) were added to another 30 mL of ultrapure water and stirred for 30 min to form Solution B. Solutions A and B were then mixed and stirred for an additional 30 min, yielding Solution C. After stirring, the mixture was transferred into a 100 mL autoclave and heated at 120 °C in an oven for 10 h. Upon completion, the autoclave was allowed to cool to room temperature. The supernatant was removed, and the precipitate was washed three times with ultrapure water and methanol, then dried in a vacuum oven at 60 °C. The resulting powder was denoted as CoFe-LDH.

Scanning electron microscopy (SEM) and energy-dispersive X-ray spectroscopy (EDS) analyses were performed using a Jeol JSM-IT700HR (Jeol, Tokyo, Japan), scanning electron microscope equipped with an Oxford Xplore 30 spectrometer. The samples were sputter-coated with gold for 45 s at a current of 10 mA, and imaging was conducted at an accelerating voltage of 3 kV. X-ray diffraction (XRD) patterns were recorded on a Rigaku MiniFlex600 diffractometer at a scanning rate of 10°/min. Fourier transform infrared (FTIR) spectra were obtained using a Thermo Fisher Scientific NICOLET iS20 spectrometer over a scanning range of 400–4000 cm^−1^ with 32 scans. X-ray photoelectron spectroscopy (XPS) analysis was carried out on a Thermo Fisher K-Alpha system (Thermo Scientific, Waltham, MA, USA) equipped with a monochromatic Al Kα X-ray source (12 kV, 6 mA) with a 400 μm spot size. Survey spectra were collected with a pass energy of 150 eV and a step size of 1 eV. Meanwhile, high-resolution spectra were acquired with a pass energy of 50 eV and a step size of 0.1 eV, using five signal accumulation cycles to achieve high signal-to-noise ratio data.

### 2.3. Experimental Method

A 5 mg aliquot of CoFe-LDH was placed into a 250 mL beaker, which was wrapped with aluminum foil to protect it from light. Subsequently, 100 mL of a prepared tetracycline (TC) solution was transferred into the beaker using a measuring cylinder. A magnetic stirrer was then activated to continuously stir the solution at a rate of 300 rpm throughout the reaction. After 10 min, 1 mL of a 0.1 mol/L peroxymonosulfate (PMS) solution was added to the reaction system. The moment of PMS addition was taken as the reaction starting point (*t* = 0). At predetermined time intervals, a fixed volume of the reaction solution was withdrawn using a micropipette and immediately transferred into a cuvette. The absorbance of TC was measured using a UV-Vis spectrophotometer at a wavelength of 352 nm, and the corresponding TC concentration after reaction was calculated based on a standard calibration curve. For the quenching experiments, two types of quenchers were separately added to the reaction system: methanol (MeOH), which quenches both ^•^OH and $SO_{4}^{•-}$, and tert-butanol (TBA), which selectively quenches ^•^OH.

### 2.4. Analytical Methods

The evolution of TC absorbance during the catalytic reaction was continuously monitored using a UV-Vis spectrophotometer at a working wavelength of 352 nm, and the obtained absorbance values were converted into concentrations using a standard calibration curve. The apparent removal efficiency of the target pollutant by the CoFe-LDH composite was systematically quantified by measuring the reduction in TC concentration during adsorption and photocatalytic degradation. The calculation formula is shown in Equation (1):

(1) $$R\;=\;\frac{C_{0}-C_{t}}{C_{0}}\;\times \;100\%$$

where *R* represents the removal efficiency of the organic pollutant, and *C*_0_ and *C*_t_ denote the initial concentration and the concentration at reaction time *t*, respectively.

The degradation of TC in the CoFe-LDH-catalyzed PMS system was fitted using linear regression. The fitting results were found to conform to a pseudo-first-order reaction kinetics model.

## 3. Results and Discussion

### 3.1. Characterization Analysis of CoFe-LDH

#### 3.1.1. SEM

**Figure 1:**
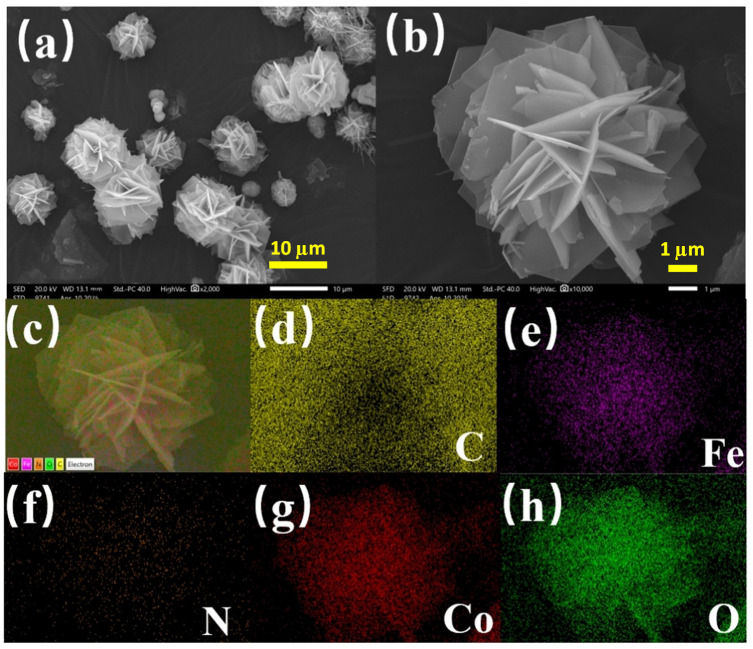
(**a**–**c**) SEM images of CoFe-LDH, (**d**–**h**) elemental distribution of CoFe-LDH.

SEM images of CoFe-LDH are presented in Figure 1. The material exhibited typical layered double hydroxide morphological characteristics. At low magnification, a three-dimensional nanoflower-like spherical structure formed by interlaced nanosheets can be observed, with a lateral size distribution within 20 µm. This structure is an ideal microstructure for CoFe-LDH materials, which can increase the specific surface area, expose more active sites, construct efficient material transport channels, eliminate internal diffusion limitations, enhance structural stability, and prevent nanoparticle aggregation. The nanosheets have smooth surfaces and well-defined edges. High-magnification images further reveal the secondary structure of the nanosheets, which are tightly interconnected via edge interactions such as hydrogen bonding or electrostatic forces, forming an open porous network. This hierarchical porous structure facilitates electrolyte penetration and exposure of active sites. In certain regions, curling or folding of the nanosheets was observed, which is likely attributable to the growth kinetics of the lamellae or interlayer stress induced by intercalated $CO_{3}^{2-}$ anions during synthesis. Moreover, the material exhibits uniform distribution with no obvious agglomeration or impurity particles, indicating well-controlled synthesis conditions. This spherical microstructure with a high specific surface area effectively promotes mass transfer during catalytic reactions and provides a stable support framework for metal active sites, thereby enhancing material performance [42]. The EDS elemental mapping confirms that CoFe-LDH is composed of Co, Fe, C, N, and O, all of which are uniformly distributed across the surface of the nanosheets.

#### 3.1.2. XRD

**Figure 2:**
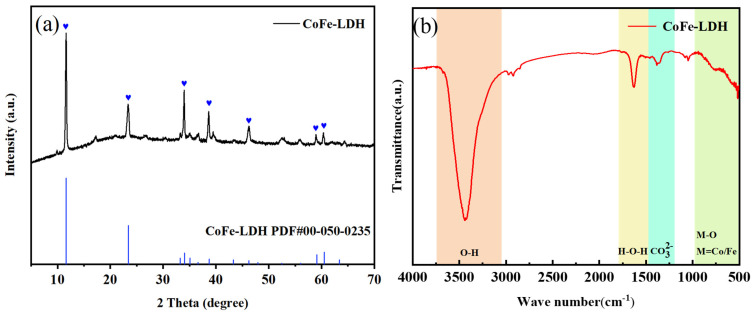
XRD (**a**) and FT-IR (**b**) of CoFe-LDH.

The XRD pattern of CoFe-LDH exhibits the typical crystal structure characteristics of a layered double hydroxide (Figure 2a). A strong and sharp diffraction peak corresponding to the (003) plane is observed at 2θ = 11°, indicating an ordered layered stacking structure with $CO_{3}^{2-}$ anions intercalated between the layers. As the angle increases, the (006) plane peak appeared near 2θ = 22°, with an intensity approximately one-third that of the (003) peak, further confirming the periodic arrangement of the layered structure. Diffraction peaks corresponding to the (012), (015), and (018) planes are observed in the 2θ range of 34–46°. These peaks are symmetric and show no splitting, suggesting that the Co and Fe ions within the layers are uniformly distributed in a hexagonal close-packed arrangement without significant lattice distortion. Clear peaks corresponding to the (110) and (113) planes are observed near 2θ = 60°. In addition, the relatively narrow full width at half maximum (FWHM) of the main peaks indicates good crystallinity and well-developed lamellar growth. The XRD pattern is in good agreement with the standard PDF card for CoFe-LDH, confirming the successful synthesis of the material [45].

#### 3.1.3. FT-IR

FT-IR spectroscopic analysis of the CoFe-LDH material reveals that the characteristic spectral features are in good agreement with the typical coordination configuration of layered double hydroxides (LDHs), particularly showing a significant correlation with the hydroxyl stretching vibration and the characteristic absorption bands of metal-oxygen bonds (Figure 2b). A broad and intense absorption band at 3440.42 cm^−1^ is assigned to the O–H stretching vibration of interlayer hydroxyl groups (–OH), and its broad profile reflects the extensive presence of a hydrogen bonding network. The absorption band at 1628.76 cm^−1^ corresponds to the H–O–H bending vibration of interlayer water molecules, further confirming the association of water molecules with the layers. The sharp and intense peak at 1383.76 cm^−1^ is attributed to the asymmetric stretching vibration of $CO_{3}^{2-}$, indicating that the interlayer anions are predominantly $CO_{3}^{2-}$. In the low-wavenumber region, the peak at 517.67 cm^−1^ is assigned to the metal–oxygen (M–O) stretching vibrations of Co–O and Fe–O within the layers, reflecting the integrity of the LDH layered framework. These FT-IR features are consistent with the layered structure of CoFe-LDH and its $CO_{3}^{2-}$ intercalation characteristics, providing chemical bonding evidence to support the structural design and performance optimization of the material [45,46].

#### 3.1.4. XPS

**Figure 3:**
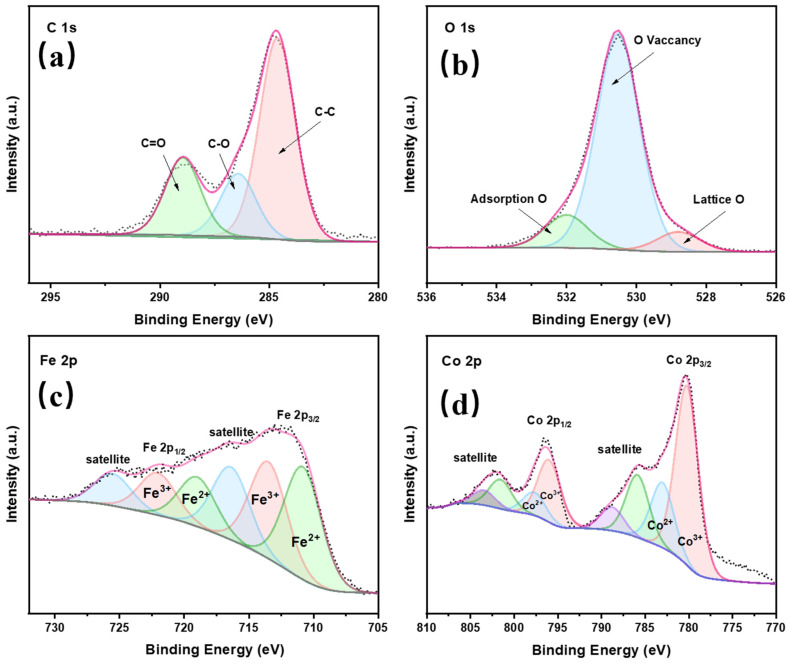
(**a**) C1s, (**b**) O1s, (**c**) Fe2p, (**d**) Co2p of CoFe-LDH.

XPS analysis confirmed the presence of four elements: C, O, Fe, and Co (Figure 3). In the O 1s spectrum, a strong characteristic peak corresponding to hydroxyl groups was observed at 531.5 eV, indicating the presence of hydroxyl groups in CoFe-LDH [47]. The other two characteristic peaks were located at binding energies of 528.86 eV and 532.1 eV, which are attributed to metal–OH and carbonate ($CO_{3}^{2-}$), respectively. The Fe 2p spectrum consisted of Fe 2p_1/2_ (722 eV) and Fe 2p_3/2_ (712.3 eV), with the presence of both Fe(III) and Fe(II). Similarly, Co 2p_1/2_ and Co 2p_3/2_ peaks appeared at binding energies of 796.2 eV and 780.4 eV, corresponding to Co(III) and Co(II), respectively.

In summary, CoFe-LDH has a three-dimensional flower-shaped layered structure, abundant surface hydroxyl groups, and mixed valence states of Co^2+^/Co^3+^ and Fe^2+^/Fe^3+^. These characteristics can improve TC mass transfer and contact, promote efficient activation of PMS, generate highly active species, and accelerate TC degradation.

### 3.2. Removal Efficiency of TC by CoFe-LDH

#### 3.2.1. Adsorption Effect of CoFe-LDH on TC

**Figure 4:**
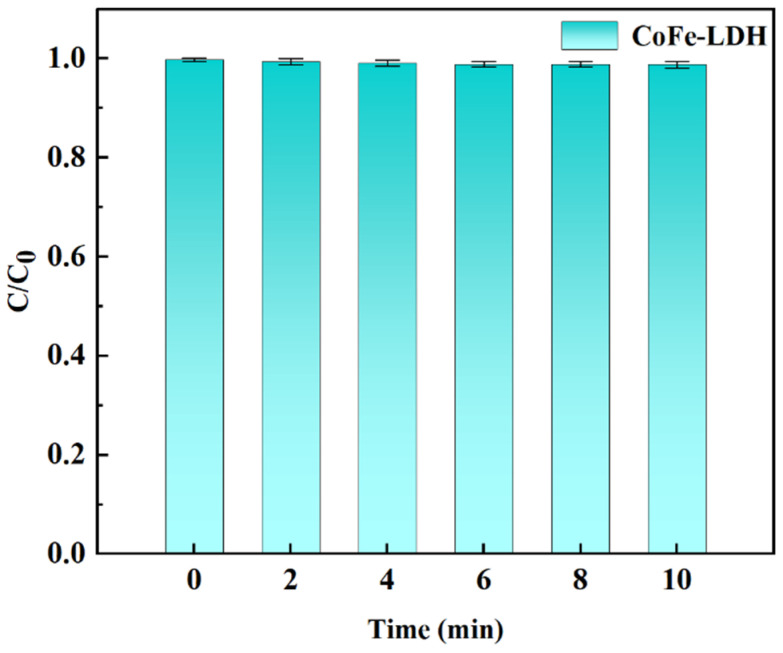
Adsorption Effect of CoFe-LDH on TC.

Prior to conducting the catalytic degradation experiments at room temperature, the adsorption performance of CoFe-LDH was evaluated. As shown in Figure 4, CoFe-LDH exhibited negligible adsorption capacity for TC, with a removal efficiency of only 1.2% within 10 min. Therefore, in subsequent experiments investigating factors influencing TC degradation, TC removal was primarily achieved by adding PMS. Based on the above adsorption behavior, a mechanical stirring step of 10 min was performed before each catalytic experiment to allow CoFe-LDH to reach adsorption equilibrium, after which PMS was introduced into the reaction system to initiate the degradation reaction.

#### 3.2.2. Degradation Effect of TC by PMS Activated by CoFe-LDH

**Figure 5:**
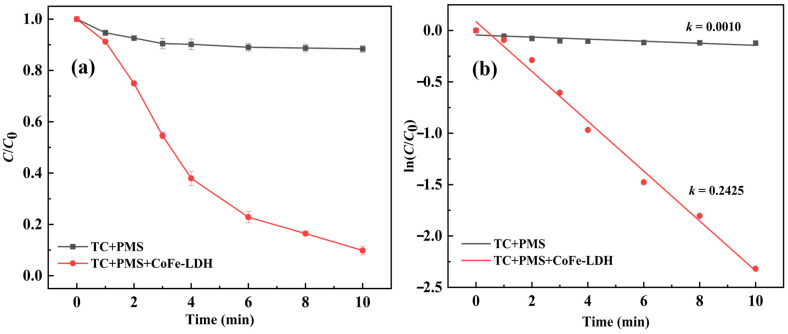
(**a**) Removal rate of TC in different systems, (**b**) Reaction rate constants in different systems.

As shown in Figure 5, in the presence of PMS alone, the degradation efficiency of TC was approximately 11% after 10 min, with the degradation trend gradually approaching a plateau. This limited degradation is primarily attributed to the inherently weak oxidation capacity of PMS. In contrast, upon the addition of CoFe-LDH and PMS, the CoFe-LDH/PMS system achieved a TC removal efficiency of 90.17% after 10 min, indicating that CoFe-LDH can effectively activate PMS to generate abundant reactive species. Kinetic fitting of TC degradation in the PMS-alone and CoFe-LDH/PMS systems revealed that the reaction rate constant of the CoFe-LDH/PMS system was 242 times higher than that of PMS alone, demonstrating that CoFe-LDH can efficiently and rapidly activate PMS for TC degradation. No leaching of Co and Fe was observed during the experiment. After three cycles, the removal rate of TC remained at 84.77% after 10 min, indicating good performance stability of the CoFe-LDH catalyst.

### 3.3. Influence Conditions of Reaction System

#### 3.3.1. Material Dosage

**Figure 6:**
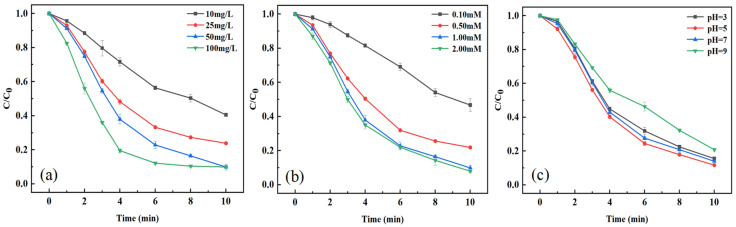
Effect of material dosage (**a**), PMS dosage (**b**), and pH (**c**).

The effect of CoFe-LDH dosage on TC degradation efficiency was systematically investigated within a concentration range of 10–100 mg/L ([PMS]_0_ = 1.00 mM, [TC]_0_ = 20 mg/L; the pH was not adjusted). As shown in Figure 6a, the degradation efficiency of TC exhibited a significant increasing trend with increasing CoFe-LDH dosage. When the catalyst dosage increased from 10 mg/L to 50 mg/L, the removal efficiency of TC within 10 min markedly improved from 40% to 90%. This enhancement is attributed to the positive correlation between the density of active sites and the amount of catalyst added. However, when the dosage was further increased to 100 mg/L, although the reaction kinetics were notably accelerated, the removal efficiency reached a plateau (remaining at approximately 90%). This phenomenon is likely due to particle agglomeration caused by catalyst overloading, thereby reducing the exposure efficiency of the active sites.

#### 3.3.2. PMS Dosage

The effect of PMS dosage on TC degradation was investigated, and the results are shown in Figure 6b ([CoFe-LDH]_0_ = 50 mg/L, [TC]_0_ = 20 mg/L, the pH is not adjusted). For a 100 mL TC solution with an initial concentration of 20 mg/L and a catalyst dosage of 50 mg/L, the TC removal efficiency increased from 53.3% to 78.14% and further to 90.17% as the PMS dosage was increased from 0.10 mM to 0.50 mM and then to 1.00 mM, accompanied by an enhanced degradation rate. However, when the PMS concentration was further increased to 2.00 mM, no significant improvement in degradation efficiency was observed compared to that at 1.00 mM. This phenomenon can be attributed to excessive radical generation induced by the high PMS dosage, leading to self-quenching reactions among reactive species. Considering both degradation performance and economic feasibility, the optimal PMS dosage was determined to be 1.00 mM.

#### 3.3.3. pH

The solution pH plays a critical role in the catalytic activation of PMS, as it significantly influences the degradation rate by altering the charge distribution states of pollutant molecules, oxidants, and catalyst surfaces. This study used hydrochloric acid and sodium hydroxide to adjust the initial pH of the reaction solution, and measured the pH using a digital pH meter. As shown in Figure 6c, when the pH gradually increased from 5.0 to 9.0, the degradation efficiency of TC progressively decreased([PMS]_0_ = 1.00 mM, [CoFe-LDH]_0_ =50 mg/L, [TC]_0_ = 20 mg/L). This is likely due to the reaction between $SO_{4}^{•-}$ and OH^−^ as the pH rises, which reduces the reaction rate [48]. At an initial pH of 3, the removal efficiency of TC in the CoFe-LDH/PMS system also declined, which can be attributed to the suppression of ≡Co active sites on the catalyst surface under strongly acidic conditions, thereby hindering the effective generation of $SO_{4}^{•-}$ and ultimately reducing the catalytic oxidation efficiency [49]. Therefore, the optimal degradation performance was achieved under weakly acidic conditions.

#### 3.3.4. Inorganic Anions

**Figure 7:**
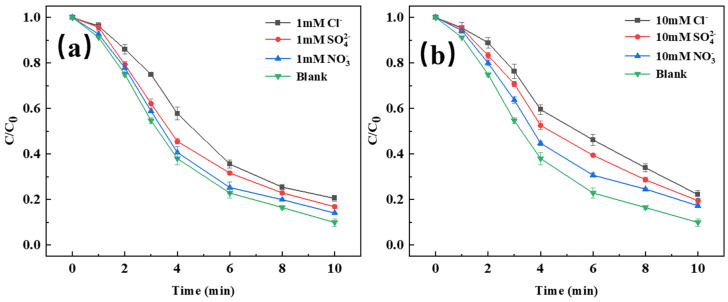
Effect of 1 mM (**a**) and 10 mM (**b**) inorganic anion on TC removal.

Inorganic anions commonly present in natural water bodies and actual wastewater can significantly affect the degradation of TC. Therefore, the effects of three anions—$SO_{4}^{2-}$, $NO_{3}^{-}$, and Cl^−^—at both low and high concentrations on TC removal were investigated, as shown in Figure 7. All three inorganic anions exhibited inhibitory effects on TC degradation, with the inhibition intensifying as the anion concentration increased. The order of inhibition strength, from highest to lowest, was Cl^−^ > $SO_{4}^{2-}$ > $NO_{3}^{-}$. Under the condition of 10 mM Cl^−^, $SO_{4}^{2-}$, $NO_{3}^{-}$ concentration, the removal rates of TC decreased to 77.88%, 80.58%, and 82.78%, respectively. When Cl^−^ was present in the solution, the degradation efficiency was considerably affected, primarily because Cl^−^ reacts with radicals to generate reactive species with weaker oxidation capacity, such as Cl^•^ and HOCl^•−^, thereby reducing the availability of $SO_{4}^{•-}$/^•^OH and suppressing the degradation of organic pollutants, leading to a decrease in TC removal efficiency [50,51]. $SO_{4}^{2-}$ does not react directly with $SO_{4}^{•-}$, but it can inhibit organic pollutant degradation in the presence of sulfate radicals. This is attributed to the effect of sulfate ions on the reduction potential of sulfate radicals—high concentrations of sulfate ions lower the reduction potential, thereby impairing degradation performance [52]. $NO_{3}^{-}$ can react with radicals in the system to generate $NO_{3}^{•}$, consequently inhibiting degradation [53].

([PMS]_0_ = 1.00 mM, [CoFe-LDH]_0_ = 50 mg/L, [TC]_0_ = 20 mg/L)

### 3.4. Universality of CoFe-LDH/PMS

#### 3.4.1. Degradation Effect of Different Target Pollutants

**Figure 8:**
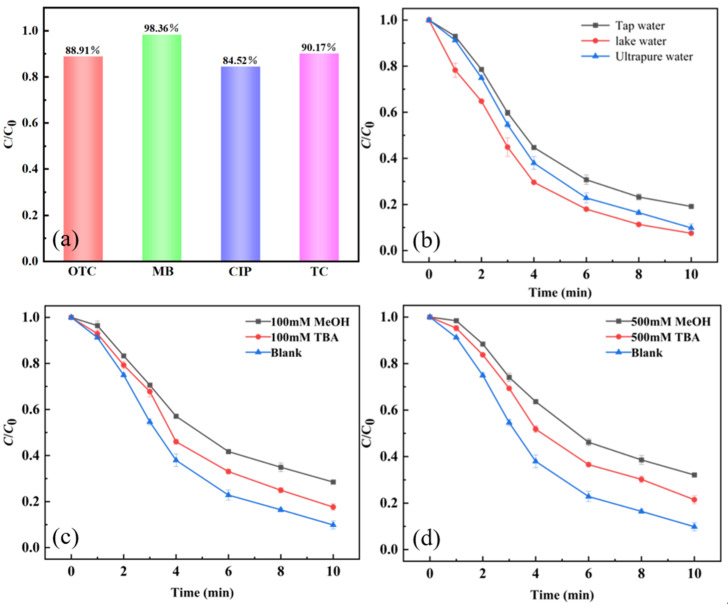
Other contaminants (**a**), different water systems (**b**), quencher 100 mM (**c**), quencher 500 mM (**d**).

To evaluate the broad-spectrum applicability of the CoFe-LDH/PMS system for the degradation of recalcitrant organic pollutants, degradation experiments were conducted using other additional categories of typical pollutants: oxytetracycline (OTC), a tetracycline antibiotic belonging to the same class as TC; methylene blue (MB), an organic dye; and ciprofloxacin (CIP), a fluoroquinolone antibiotic. The results are shown in Figure 8a. Within a 10 min degradation period, the degradation efficiency of OTC (88.91%) by the CoFe-LDH/PMS system was comparable to that of TC, while the system exhibited the highest degradation efficiency for MB (98.36%). Although the degradation efficiency for CIP was slightly lower, it still exceeded 84%. The CoFe-LDH maintained good catalytic activity across the different pollutant systems, with its active sites demonstrating effective oxidation toward organic compounds of varying structures. These results confirm the broad-spectrum applicability of the CoFe-LDH/PMS system for the treatment of recalcitrant organic pollutants.

([PMS]_0_ = 1.00 mM, [CoFe-LDH]_0_ = 50 mg/L, the pH is not adjusted)

#### 3.4.2. Degradation Effect in Actual Water

To investigate the catalytic performance of the catalyst in different water matrices, tap water and Dongting Lake water were used as reaction media. As shown in Figure 8b, the CoFe-LDH/PMS reaction system maintained relatively high TC degradation efficiency even under simulated tap water (Changsha, China) and Dongting Lake water (China). In tap water, a slight inhibitory effect was observed, leading to a decrease in TC degradation efficiency (80.86%). This may be attributed to the coexistence of various organic molecules, inorganic salts, and other unknown factors in actual wastewater, which can scavenge the reactive radicals generated in the CoFe-LDH/PMS system [54]. In contrast, Dongting Lake water enhanced TC degradation efficiency (92.48%). This enhancement may be associated with the presence of certain components in this water matrix that facilitate the activation of persulfate. These components could act as electron transfer mediators or catalysts, thereby enhancing the overall oxidation efficiency and consequently improving the degradation performance.

### 3.5. Analysis of Degradation Mechanism

To identify the reactive oxygen species (ROS) responsible for TC degradation, radical quenching experiments were conducted to determine the types of ROS involved in the CoFe-LDH/PMS reaction system. In this experiment, methanol (MeOH) was used as a quencher for both ^•^OH and $SO_{4}^{•-}$, while tert-butanol (TBA) was used specifically to quench ^•^OH. The experimental results are shown in Figure 8c,d. As presented in Figure 8c, the addition of 100 mM MeOH and TBA resulted in an inhibitory effect on TC degradation, with MeOH exhibiting a stronger inhibition than TBA. This indicates that both ^•^OH and are involved in TC removal by the CoFe-LDH/PMS system [36,55]. As shown in Figure 8c,d, MeOH exhibited a stronger inhibitory effect on TC degradation than TBA. Therefore, the contribution rate of $SO_{4}^{•-}$ to TC degradation is greater than that of ^•^OH. The Co and Fe in the CoFe-LDH structure serve as key active centers to activate PMS and generate $SO_{4}^{•-}$. $SO_{4}^{•-}$ react with hydroxide ions to generate ^•^OH. Co and Fe ions form a cycle through electron transfer, accelerating the kinetics of the catalytic reaction. Low valence Co (II) or Fe (II) reacts with PMS to generate high valence Co (III) or Fe (III) and key active species. High valence metal ions can be reduced back to their low valence state through interactions with each other or organic compounds in the system, forming a cycle and continuously catalyzing.

## 4. Conclusions

(1) A three-dimensional flower-like CoFe-LDH material was synthesized via a hydrothermal method. Its crystal structure and chemical composition were confirmed using multiple characterization techniques, including SEM, XRD, FTIR, and XPS.
(2) CoFe-LDH exhibited negligible adsorption capacity for TC. However, upon the addition of PMS, the degradation efficiency of TC reached 90.17% within 10 min. Kinetic analysis revealed that the apparent reaction rate constant for TC degradation in the CoFe-LDH/PMS system was 242 times higher than that in the PMS-alone system.
(3) The CoFe-LDH/PMS system achieved optimal performance under weakly acidic conditions, with the optimal dosages of CoFe-LDH and PMS being 50 mg/L and 1.00 mM, respectively. The presence of interfering anions such as Cl^−^, $NO_{3}^{-}$, and $SO_{4}^{2-}$ inhibited the degradation process.
(4) The CoFe-LDH/PMS system also demonstrated high efficiency in degrading other organic pollutants, including OTC, MB, and CIP. Its performance was minimally affected by matrix interference from lake water and tap water, confirming its broad applicability.
(5) Radical quenching experiments confirmed the involvement of ^•^OH and $SO_{4}^{•-}$ in the reaction, with $SO_{4}^{•-}$ playing a more dominant role in TC degradation than ^•^OH.

## Data Availability

The original contributions presented in this study are included in the article. Further inquiries can be directed to the corresponding authors.
